# Loss of NKG2D in murine NK cells leads to increased perforin production upon long‐term stimulation with IL‐2

**DOI:** 10.1002/eji.201948222

**Published:** 2020-02-20

**Authors:** Daniela Prinz, Klara Klein, Julia List, Vanessa M. Knab, Ingeborg Menzl, Nicoletta Leidenfrost, Gerwin Heller, Bojan Polić, Eva Maria Putz, Agnieszka Witalisz‐Siepracka, Veronika Sexl, Dagmar Gotthardt

**Affiliations:** ^1^ Institute of Pharmacology and Toxicology University of Veterinary Medicine Vienna Austria; ^2^ Institute of Internal Medicine I Medical University Vienna Austria; ^3^ Department of Histology and Embryology, Faculty of Medicine University of Rijeka Rijeka Croatia; ^4^ St. Anna Children's Cancer Research Institute (CCRI) Medical University of Vienna Vienna Austria

**Keywords:** NKG2D, NK cell, perforin, *Ncr1*‐iCre^Tg^, tumor surveillance

## Abstract

NK cells are innate lymphocytes responsible for lysis of pathogen‐infected and transformed cells. One of the major activating receptors required for target cell recognition is the NK group 2D (NKG2D) receptor. Numerous reports show the necessity of NKG2D for effective tumor immune surveillance. Further studies identified NKG2D as a key element allowing tumor immune escape. We here use a mouse model with restricted deletion of NKG2D in mature NKp46^+^ cells (NKG2D^ΔNK^). NKG2D^ΔNK^ NK cells develop normally, have an unaltered IFN‐γ production but kill tumor cell lines expressing NKG2D ligands (NKG2DLs) less efficiently. However, upon long‐term stimulation with IL‐2, NKG2D‐deficient NK cells show increased levels of the lytic molecule perforin. Thus, our findings demonstrate a dual function of NKG2D for NK cell cytotoxicity; while NKG2D is a crucial trigger for cytotoxicity of tumor cells expressing activating ligands it is also capable to limit perforin production in IL‐2 activated NK cells.

## Graphical abstract

NKG2D has a dual function in NK cell cytotoxicity; while NKG2D is a crucial trigger for cytotoxicity against tumor cells expressing activating ligands, it limits perforin production in IL‐2 activated NK cells. The increased perforin levels are not linked to an altered activation of the JAK/STAT pathway.

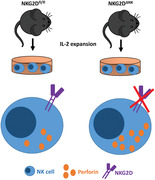

## Introduction

NK cells are innate lymphoid cells known for their potential to kill pathogen‐infected and transformed cells. NK cells eliminate tumor cells mainly through the release of lytic granules containing perforin and granzymes and via secretion of pro‐inflammatory cytokines such as IFN‐γ and TNF‐α [1]. This antitumor activity is controlled by balancing inputs from activating and inhibitory receptors [2].

One well‐studied activating receptor of NK cells in mice and men is the NKG2D receptor. NKG2D is mainly expressed by NK cells and NKT cells, but can also be found on the surface of γδ T cells and subsets of activated T cells. Ligands of NKG2D comprise stress‐induced MHC class I (MHC‐I) related proteins. There are two types of human NKG2DLs; MHC‐I chain‐related protein A/B proteins (MICA/B) and UL16‐binding proteins (ULBP‐family). In contrast, mice display three groups of proteins that possess the ability to activate NKG2D, namely murine ULBP transcript 1 (MULT‐1), histocompatibility antigen 60 family of molecules (H60a, b, and c) and the retinoic acid early‐inducible 1 family of proteins (RAE‐1α, β, γ, δ, ε) [3]. Expression of NKG2DLs renders cells visible for NK cells and NKG2D stimulation can overrule inhibitory signals [4, 5]. Numerous reports have provided solid evidence for the importance of NKG2D in tumor surveillance and show that *Klrk1* ^−/−^ (the gene encoding NKG2D) mice are more susceptible to tumor development than control mice [5, 6, 7, 8]. Despite these reports demonstrating impaired tumor surveillance in NKG2D‐deficient mouse models, NKG2DL expression in tumor‐bearing patients has been associated with either good or poor prognosis depending on the tumor entity [9]. The fact that tumor cells evade NKG2D‐dependent elimination by diverse mechanisms such as ligand shedding or release of cytokines downregulating NKG2D adds another layer of complexity [10]. In addition, constitutive NKG2D stimulation provokes receptor internalization and thereby decreases NK cell effector functions. NK cells from mice ubiquitously expressing RAE‐1ε fail to express NKG2D on the cell surface and show an impaired in vivo cytotoxic capacity [11]. Incubation of NK cells with NKG2DL‐bearing tumor cells for 72 h induces a profound downregulation of NKG2D and defective Ca^2+^ mobilization upon re‐stimulation [12]. A follow‐up study uncovered that constitutive NKG2D stimulation leads to cross‐tolerance of other NK cell activating receptors. An impaired Ca^2+^ mobilization by multiple NK cell receptors is discussed as the underlying mechanism [13]. Jelenčić et al. supported these observations providing evidence that NKG2D sets an activation threshold for NKp46 activation in early NK cell development [14]. An elegant study by Deng et al. demonstrated that shed ligands prevent a prolonged NKG2D‐stimulation and enhance tumor surveillance. They showed that intra‐tumoral myeloid cells express NKG2DLs on their surface and thereby contribute to NKG2D‐dependent NK cell desensitization [15]. They also described the ligand RAE‐1ε as constitutively expressed on LN endothelial cells and a significant upregulation of RAE‐1ε on tumor endothelial cells. The interplay between NKG2D and endothelial RAE‐1ε modulates the responsiveness of NK cells under steady state conditions and in the tumor microenvironment [16]. Interestingly, chronic stimulation with the NKG2DL RAE‐1 does not impair NK cell functionality in the context of viral infection [17]. This diverse and complex role of NKG2D in NK cell functionality stresses the need for a better understanding of underlying regulatory mechanisms to harness NK cell cytotoxic potential for tumor immunotherapy.

Studies so far have investigated effects of constitutive NKG2D deficiency when absent in the entire organism. We here now investigate the consequences of NKG2D deletion in mature NK cells for NK cell‐mediated tumor surveillance.

## Results

### NK cell‐specific NKG2D deletion does not alter NK cell maturation and surface receptor expression

**Figure 1:**
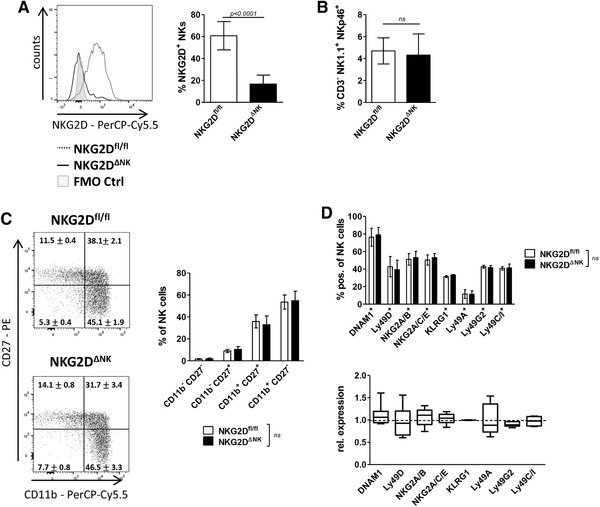
NK cell maturation and surface receptor expression is unaltered upon NK cell‐specific deletion of NKG2D. (A) The deletion efficiency of NKG2D in splenic NKG2D^ΔNK^ CD3^−^NKp46^+^NK1.1^+^ NK cells was analyzed by flow cytometry. Left panel: representative histogram; right panel: bar graph showing percentage of NKG2D^+^ cells gated on CD3^−^NK1.1^+^NKp46^+^ lymphocytes. (B) Percentage of NK cells in the spleen defined as CD3^−^NK1.1^+^NKp46^+^ is shown. (C) Maturation stages of splenic NKG2D^ΔNK^ and NKG2D^fl/fl^ CD3^−^NKp46^+^NK1.1^+^ NK cells were analyzed using CD11b and CD27 expression by flow cytometry. Representative dot plots are shown. (D) Splenocytes of NKG2D^ΔNK^ and NKG2D^fl/fl^ control mice were analyzed for surface receptor expression gated on CD3^−^NK1.1^+^NKp46^+^ cells via flow cytometry. Upper panel shows percentage of NK cells stained positive for indicated surface receptors. Lower panel shows MFI of surface receptors on NKG2D^ΔNK^ NK cells relative to MFI on NKG2D^fl/fl^ NK cells. Bar graphs in (A) show mean ± SD (*n* = 23 mice per genotype pooled from four independent experiments). Bar graphs in (B) and (C) show mean ± SD (*n* = 12 mice per genotype pooled from three independent experiments). (D) Bar graph show mean ± SD (*n* = 6 mice per genotype pooled from two independent experiments). Plot shows the median MFI ± interquartile range of expression of indicated receptors in NKG2D‐deficient NK cells relative to control. Two‐sided unpaired *t*‐test was performed for all experiments. Gating strategies are shown in Supporting Information Fig. 6A and B.

To study the consequences of NKG2D deficiency in the NK cell compartment, we generated conditional NKG2D KO mice by crossing *Klrk1^fl/fl^* mice [18] to *Ncr1‐iCre^Tg^* mice [19]. In these mice, the *Klrk1* gene (encoding for NKG2D) is efficiently deleted in all NKp46^+^ cells (*Klrk1^fl/fl^ Ncr1‐iCre^Tg^*, hereafter termed as NKG2D^ΔNK^; Fig. 1A). Percentages of NK cells in the spleen and BM of NKG2D^ΔNK^ mice did not differ from NKG2D^fl/fl^ littermates (Fig. 1B; Supporting Information Figs. 1A, 6A and C). We investigated the impact of NK cell‐intrinsic loss of NKG2D on NK cell development, which takes place in the BM. Analysis of NK cell precursors (NKPs: CD3^−^CD19^−^Ter119^−^Gr1^−^(Lin^−^) CD122^+^NK1.1^−^NKp46^−^), immature NK cells (iNKs: Lin^−^CD122^+^NK1.1^+^NKp46^−^), and mature NK cells (mNKs: Lin^−^CD122^+^NK1.1^+^NKp46^+^) in the BM did not reveal any significant differences between genotypes (Supporting Information Figs. 1B and 6C).

While NK cell development takes place in the BM, maturation of NK cells occurs in the spleen, where four maturation stages can be distinguished by the expression of CD27 and CD11b [20]. We found that NK cell maturation in the spleen was unaltered upon NKG2D deletion (Fig. 1C).

A delicate balance between activating and inhibitory receptors controls NK cell activity. Flow cytometry analysis of a panel of NK cell receptors did not reveal any differences in percentage of positive cells or level of expression (measured by MFI) [DNAM1, Ly49D, NKG2A, NKG2A/C/E, KLRG1, Ly49A, Ly49G2, and Ly49C/I] (Fig. 1D; Supporting Information Fig. 6B). We conclude that deletion of NKG2D in mature NK cells does not affect NK cell development, maturation, and surface receptor expression.

### NKG2D‐deficient NK cells respond normally to cytokine and activating receptor stimulation

**Figure 2:**
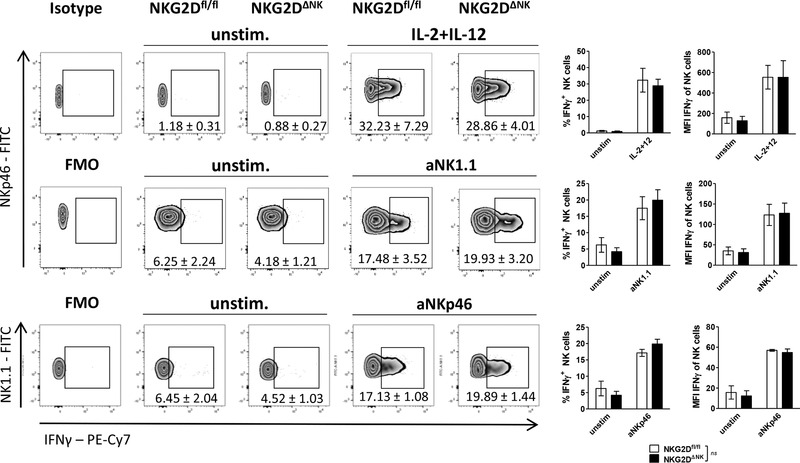
NKG2D‐deficient NK cells have unaltered IFN‐γ production upon cytokine and activating receptor stimulation. Freshly isolated splenocytes of NKG2D^ΔNK^ or NKG2D^fl/fl^ mice were incubated with either IL‐2^+^IL‐12, immobilized mABs, or media alone (unstim.) as indicated. Cells were stimulated for 4 h and percentage of IFN‐γ^+^ CD3^−^NKp46^+^NK1.1^+^ NK cells and MFI of all NK cells was analyzed via flow cytometry. Cells stimulated with aNK1.1 mAB were gated on CD3^−^NKp46^+^. In contrast, aNKp46 stimulated splenocytes were gated on CD3^−^NK1.1^+^ cells. Results indicate mean ± SD (*n* = 6–10 mice per genotype pooled from two to three independent experiments; unpaired two‐sided *t‐*test). The gating strategy is shown in Supporting Information Fig. 6A.

NK cells are potent producers of inflammatory cytokines contributing to the complex crosstalk of immune cells and triggering recruitment of other cells to the tumor microenvironment [2]. To investigate whether the expression of IFN‐γ is affected in the presence or absence of NKG2D, we stimulated splenocytes with NK cell activating cytokines or by receptor crosslinking. Neither stimulation with IL‐2+IL‐12 nor crosslinking with anti‐NK1.1 (aNK1.1), aNKp46, or aDNAM1 antibodies in different concentrations induced changes in the IFN‐γ production of NKG2D‐deficient NK cells compared to control cells (Fig. 2; Supporting Information Fig. 2A–C).

### IL‐2 expanded NKG2D‐deficient NK cells produce significantly more perforin

**Figure 3:**
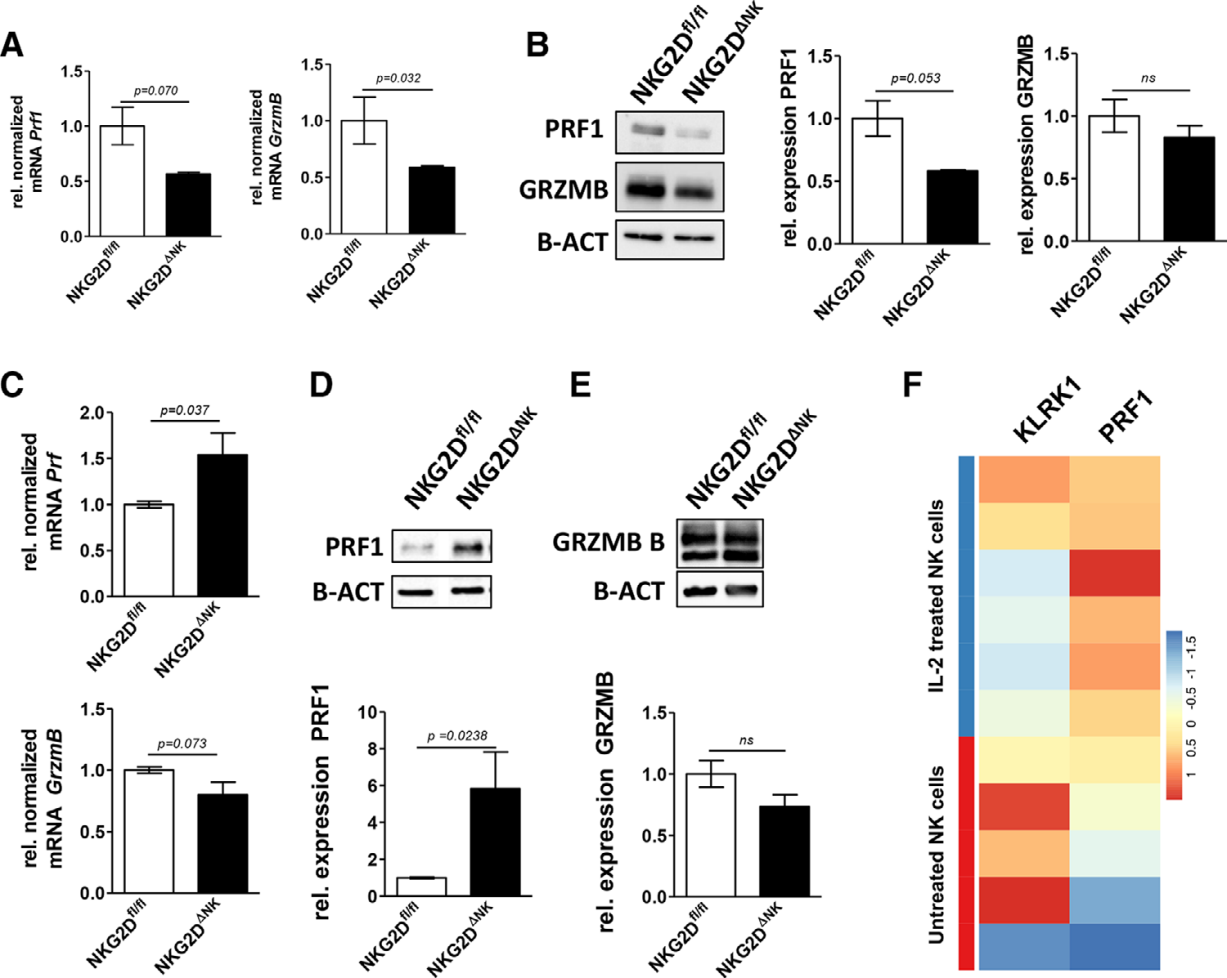
NKG2D‐deficient NK cells produce significantly more perforin upon stimulation with IL‐2. (A) mRNA expression and (B) protein levels of perforin, granzyme B, and β‐actin as loading control of ex vivo derived and sorted splenic NK cells (MACS enriched for DX5^+^ and flow cytometric sorted for CD3^−^NK1.1^+^NKp46^+^) were analyzed. Results in (A) represent expression of mRNA in NKG2D‐deficient NK cells relative to controls shown as mean ± SD (four mice were pooled per experiment per genotype; blot shows data pooled from two independent experiments; unpaired two‐sided *t*‐test). Results in (B) show mean ± SD (representative blot of two independent biological replicates pooled from four mice each and representative of two independent experiments is shown; individual blots were analyzed and expression levels were normalized to values from NKG2D^fl/fl^ NK cells; unpaired two‐sided *t‐*test.) (C) NK cells were MACS‐enriched from whole splenocytes and expanded in vitro for 7 days and perforin and granzyme B mRNA expression was analyzed using qRT‐PCR. Bars represent mean ± SD (four mice per genotype were analyzed in four independent experiments; results represent data pooled from all four experiments; unpaired two‐sided *t‐*test; relative expression levels were normalized to NKG2D^fl/fl^ controls). (D) Perforin and (E) granzyme B protein levels were determined relative to β‐actin by western blot. Bars represent mean ± SD (representative blot of three independent biological replicates pooled from two to four mice each is shown; individual blots were analyzed and expression levels were normalized to NKG2D^fl/fl^; unpaired two‐sided *t‐*test). (F) Human NK cell microarray from the online available dataset (GSE12198) was analyzed for NKG2D (*KLRK1*) and PRF1 expression. Data are shown as scaled log_2_ expression values and range from blue (low expression) to red (high expression).

NK cells exert their cytolytic action mainly through the release of perforin and granzymes. Ex vivo sorted NKG2D‐deficient NK cells showed reduced perforin and granzyme B mRNA and lower perforin protein levels while granzyme B protein production was unaltered (Fig. 3A and B; Supporting Information Figs. 2D and 6A). Considering the potential of NK cells to be used as a tumor immunotherapy upon expansion in vitro, we investigated the effect of NKG2D deletion on in vitro cultured NK cells. We confirmed NKG2D deletion in IL‐2 expanded NK cells (Supporting Information Fig. 3A) but detected elevated mRNA expression of perforin but not granzyme B (Fig. 3C) in the absence of NKG2D. In line, we saw significantly enhanced perforin expression on protein level (Fig. 3D) but no difference in granzyme B production (Fig. 3E). Microarray data from human NK cells stimulated for 7 days with IL‐2 were compared to naïve NK cells and showed likewise an inverse association of NKG2D and perforin expression upon IL‐2 treatment (Fig. 3F).

### NKG2D‐deficient NK cells show an unaltered activation of the JAK/STAT signaling pathway

**Figure 4:**
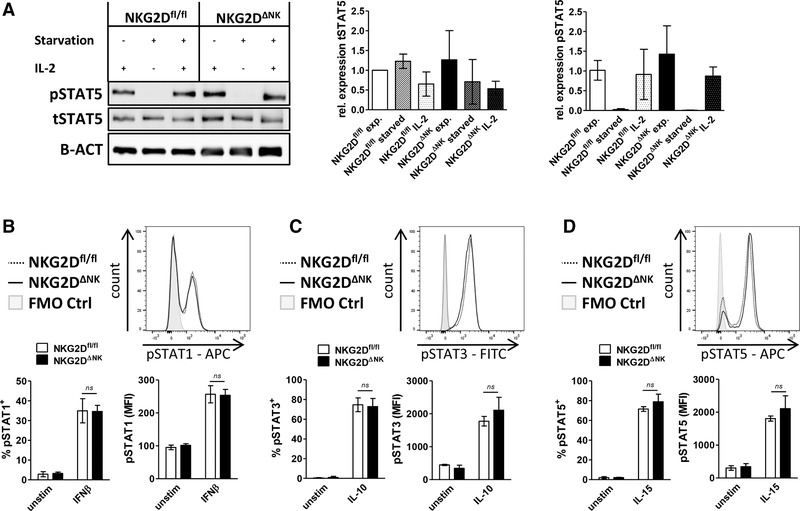
Activation of the JAK/STAT signaling pathway is unaltered in NKG2D‐deficient NK cells. (A) Activation of STAT5 was assessed in IL‐2 expanded splenic NK cells. Bar graphs show quantification of protein expression of total STAT5 or pSTAT5 expression normalized to the loading control (β‐actin) and relative to NKG2D^fl/fl^ NK cells. A representative blot of two independent experiments is shown. Bars represent mean ± SD (representative blot of two independent biological replicates pooled from two to four mice each is shown; individual blots were analyzed and expression levels were normalized to NKG2D^fl/fl^; unpaired two‐sided *t‐*test). (B–D) Splenocytes were isolated and stimulated for 20 min with IL‐15, IL‐10, and IFN‐β to activate STAT5, STAT3, and STAT1, respectively. Histograms show representative results of stimulated NK cells. Bar graphs indicate percentage of positively stained NK cells and MFI of the STAT‐positive population. Results represent mean ± SD (*n* = 4 mice per genotype pooled from two independent experiments; unpaired *t‐*test).

As the expression of perforin and granzyme B is regulated by IL‐2‐STAT5 signaling [21], we determined STAT5 activation in expanded NK cells. We did not detect a difference in total STAT5 protein level as well as in pY‐STAT5 indicative for STAT5 activation between the genotypes (Fig. 4A). Other members of the JAK/STAT family are also involved in the regulation of perforin expression [22, 23, 24] and STAT3 has been reported to control NKG2D expression [25]. We therefore analyzed activation and expression levels of the STAT family members in freshly isolated splenocytes or IL‐2 expanded NK cells after stimulation for 20 min with IFN‐β, IL‐15, or IL‐10. Activation of STAT1, STAT3, and STAT5 was analyzed via intracellular phospho‐flow staining. We did not observe any difference in STAT activation between the genotypes (Fig. 4B–D; Supporting Information Fig. 3B). In line, we observed comparable levels of p‐STAT1, p‐STAT3, and p‐STAT4 in in vitro expanded NK cells by immune blotting (Supporting Information Fig. 3C–E). We conclude that NKG2D^ΔNK^ and NKG2D^fl/fl^ NK cells have a comparable ability to induce JAK/STAT signaling upon stimulation.

### Enhanced perforin levels in NKG2D^ΔNK^ NK cells do not result in increased cytolytic activity in vitro

**Figure 5:**
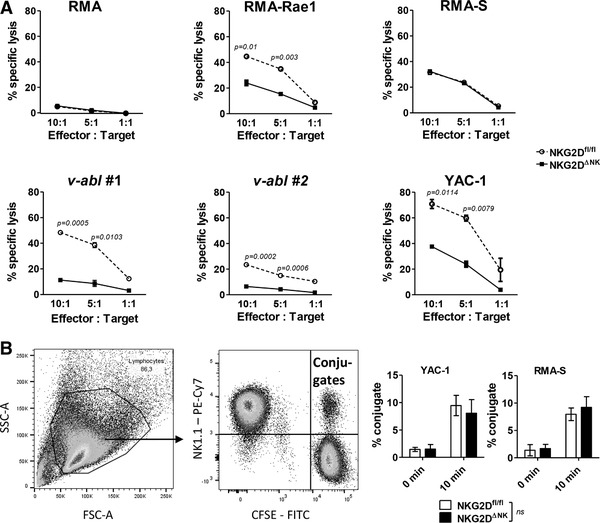
In vitro cytotoxicity of NKG2D^ΔNK^ NK cells is comparable to NKG2D^fl/fl^ controls. NK cells were MACS enriched from splenocytes and expanded in vitro for 7 days. (A) The cytotoxic capacity of IL‐2 expanded NKG2D^ΔNK^ and NKG2D^fl/fl^ NK cells was tested in a flow cytometry based assay against RMA cells as negative control, RMA‐Rae1, YAC‐1, and two in house generated *v‐abl* ^+^ cell lines as NKG2DL‐expressing NK cell targets and target cells killed NKG2D‐independently namely RMA‐S, at the indicated effector‐to‐target ratios. Graphs show mean ± SD from one representative experiment of two to three independent experiments with two to four mice per experiment and two technical replicates. (B) Conjugate formation assays were performed using target cell lines YAC‐1 and RMA‐S. Dot plot shows the gating strategy. Bar graphs show mean ± SD of conjugates upon 10 min incubation (*n* = 4 samples pooled from two independent experiments). Gating strategy for cytotoxicity assays is shown in Supporting Information Fig. 6D. Unpaired two‐sided *t*‐test was performed for experiments in (A) and (B).

As NKG2D‐deficient NK cells produce more perforin but lack a crucial activating receptor on their surface, we investigated their killing capacity in in vitro cytotoxicity assays. High dose IL‐2 culture leads to altered surface receptor expression of NK cells [26], which may interfere with cytotoxicity. We thus first analyzed IL‐2 expanded NK cells for a broad range of activating and inhibitory surface receptors by flow cytometry but did not detect any major changes between the two genotypes with exception of NKG2D (Supporting Information Figs. 4A and 6B). Leukemia and lymphoma target cell lines for cytotoxicity assays were selected based on the expression of the NK cell activating ligands RAE‐1 and MULT‐1 (detected via NKG2D) and CD155 (detected via DNAM1). The expression of these ligands varies between the different tumor cell lines with RMA‐S being the only cell line completely lacking NKG2DLs (Supporting Information Fig. 4B). The selected cell lines were CFSE‐labeled and co‐incubated with IL‐2 expanded NK cells for 4 h. RMA cells are not targeted by NK cells and served as a negative control. RMA‐RAE1 and YAC‐1 cells and two different *v‐abl* cell lines (labeled *v‐abl* #1 and *v‐abl* #2) expressing NKG2DLs to varying degrees, were killed less efficiently by NKG2D^ΔNK^ NK cells. In contrast, despite enhanced perforin levels, no difference was observed in the lysis of the TAP‐deficient RMA‐S cells lacking NKG2DLs (Fig. 5A; Supporting Information Fig. 6D). Effective killing of tumor cells requires the formation of an immunological synapse between NK cells and their targets. To verify if NKG2D deficiency in NK cells interferes with adhesion to the target cells, we performed conjugate formation assays. We found comparable conjugate formation irrespective of NKG2D expression with both YAC‐1 and RMA‐S target cells (Fig. 5B; Supporting Information Fig. 6E). NKG2D is crucial for the elimination of NKG2DL positive target cells but our results indicate that elevated perforin levels in NKG2D‐deficient NK cells do not translate into enhanced killing of NKG2DL negative target cells in vitro.

### NKG2D^ΔNK^ mice show an increased rejection of B2m^−/−^ splenocytes in vivo

**Figure 6:**
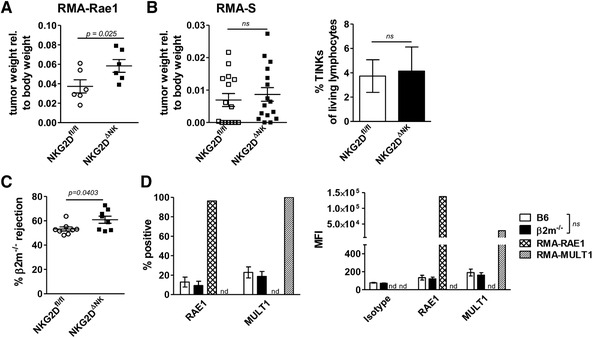
NKG2D^ΔNK^ mice are superior in rejecting B2m^−/−^ splenocytes in vivo. Tumor weight of (A) RMA‐Rae1 and (B) RMA‐S injected mice was analyzed relative to their body weight. Tumor infiltrating NK cells (TINKs) were analyzed by gating on CD3^−^NK1.1^+^NKp46^+^ lymphocytes from digested tumors. (C) NKG2D^ΔNK^ animals and NKG2D^fl/fl^ littermates were injected intravenously with a 1:1 mix of CSFE^lo^ labeled B2m^−/−^ and CSFE^hi^ labeled WT splenocytes. Recovered CSFE^hi^ and CSFE^lo^ cells from spleens of injected mice were analyzed via flow cytometry 16 h after injection. Gating strategy is shown in Supporting Information Fig. 6E. (D) RAE1 and MULT‐1 expression on B2m^−/−^ and C57BL/6N splenocytes was analyzed via flow cytometry. RMA‐Rae1 and RMA‐Mult1 cell lines served as positive control. (A) Graph shows mean ± SD (*n* = 6 mice per genotype representative of three experiments with three mice per experiment; two‐sided unpaired *t‐*test). (B) Graph shows mean ± SD (*n* = 9 mice per genotype, pooled from two independent experiments; two‐sided unpaired *t‐*test). Graph in (C) shows mean ± SD (*n* = 8 mice per genotype, pooled from two independent experiments; two‐sided unpaired *t‐*test). (D) Bar graphs represent mean ± SD (*n* = 3 mice per genotype; representative of two independent experiments, two‐sided unpaired *t‐*test).

In vitro cytotoxicity assays are a well‐established tool to assess NK cell killing capacity but fail to recapitulate the complex situation of the tumor microenvironment in vivo. We therefore investigated the consequences of NKG2D deletion in mature NK cells in an in vivo setting. As proof of principle, we injected RMA‐RAE1 cells subcutaneously into both flanks of NKG2D^ΔNK^ and NKG2D^fl/fl^ littermates. As expected, mice with NK cell‐specific deletion of NKG2D showed decreased tumor surveillance leading to an increased tumor burden (Fig. 6A). Next, we challenged mice with the NKG2DL‐negative and MHC‐I‐deficient tumor cell line RMA‐S. Tumor burden as well as percentage of tumor infiltrating NK cells (TINKs) was comparable between NKG2D^ΔNK^ and NKG2D^fl/fl^ mice (Fig. 6B) reflecting the in vitro cytotoxicity assay (Fig. 5A). We also performed subcutaneous tumor cell injections using an in‐house generated leukemic cell line *v‐abl^+^* #1. Although *v‐abl^+^* tumor cells express NKG2DLs and were therefore killed less efficiently by NKG2D^ΔNK^ NK cells in vitro, we found no differences in in vivo tumor surveillance nor in the number of tumor infiltrating NK cells comparing NKG2D^ΔNK^ and NKG2D^fl/fl^ mice (Supporting Information Fig. S5A). Intrigued by the discrepancy between the in vitro and in vivo NK cell surveillance of *v‐abl^+^* cells, we injected newborn NKG2D^ΔNK^ and NKG2D^fl/fl^ littermates with the Abelson Murine Leukemia Virus (AMuLV). This model system enables studying a slowly developing oligoclonal pro‐B cell leukemia and closely mirrors human disease [27, 28]. There was no difference in neither overall survival of the mice nor in the disease phenotype assessed by spleen to body weight ratio (Supporting Information Fig. 5B).

In addition, we performed an in vivo B2m^−/−^ splenocyte rejection assay. In this model, naïve splenocytes from B2m^−/−^ mice (lacking surface expression of MHC‐I molecules and therefore being detected by NK cells via missing self‐recognition) and WT animals were labeled with CFSE^lo^ and CFSE^hi^, mixed in a 1:1 ratio, and injected intravenously into NKG2D^ΔNK^ and NKG2D^fl/fl^ animals. In vivo rejection capacity was calculated from the ratio of B2m^−/−^ to WT splenocytes retrieved from the spleens of injected mice after 16 h. As shown in Fig. 6C, NKG2D^ΔNK^ mice show a slightly but significantly enhanced rejection capacity compared to NKG2D^fl/fl^ littermates. As naïve cells were reported to express low levels of NKG2DL under steady state conditions [15, 16], we analyzed NKG2DL expression on splenocytes from B2m^−/−^ and WT mice. Expression of RAE‐1 and MULT‐1 on splenic cells from both genotypes were indeed observed at low levels in a comparable manner (Fig. 6D).

In summary, we show that the absence of NKG2D on mature NK cells leads to an enhanced perforin production upon expansion in IL‐2. Although our finding does not translate into enhanced killing capacity of tumor cells in vitro and in vivo, we observed a slightly increased capacity of NKG2D^ΔNK^ mice to reject B2m^−/−^ splenocytes.

## Discussion

We here report that loss of NKG2D in mature NK cells does not alter NK cell numbers, development, and maturation. Our mouse model deletes only in mature NKp46^+^ NK cells whereas a complete KO mouse was used in previous studies. This may explain the discrepancy between our findings and previously published reports. Our finding opposes previous studies reporting a role of NKG2D in NK cell development [18, 29] but mirrors the findings from Guerra et al. showing unaltered NK cell developmental stages in the BM [8].

NK cells exert their cytotoxic actions via release of cytolytic granules or secretion of pro‐inflammatory cytokines including IFN‐γ. Previous studies reported enhanced IFN‐γ expression in NKG2D^−/−^ NK cells [14, 15, 18]. Our data are in line with the report from Jelenčić et al. [14], showing that NKG2D sets the activation threshold for NKp46 early in NK cell development, while deletion of NKG2D in mature NKp46^+^ cells does not impact the NKp46‐mediated IFN‐γ response. In addition, we did not observe any differences in IFN‐γ production upon stimulation with cytokines or crosslinking of NK1.1 and/or DNAM1.

NKG2D‐deficient NK cells show decreased mRNA levels of both perforin and granzyme B ex vivo. While the reduced mRNA levels translate to a decreased protein expression of perforin ex vivo, we did not detect a significant reduction in granzyme B protein expression using both western blot and intracellular flow cytometry staining. Intriguingly, we consistently detected an enhanced perforin production in NKG2D‐deficient NK cells upon IL‐2 expansion for 7 days, while again expression of granzyme B remained unaltered. RNA‐Seq results from NKG2D^−/−^ NK cells published by Jelenčić et al. [14] indicate as well an inverse association between NKG2D and perforin expression. In contrast to naïve NK cells, we observed consistently enhanced perforin mRNA levels after expansion in IL‐2. This increase in mRNA expression then further led to an increase in the protein level pointing toward an altered transcriptional regulation. Our finding is further supported by a human data set, where NKG2D and perforin show an inverse expression pattern in NK cells upon long‐term stimulation with IL‐2. As the IL‐2Rβ/STAT5 signaling pathway directly induces the Prf1 gene expression [30, 31], we first studied the activation and expression of STAT5. However, we did not detect differences in STAT5 levels or STAT5 activation (p‐STAT5) nor of other STAT family members involved in the regulation of perforin such as STAT1, STAT3, or STAT4.

As our findings are restricted to increased perforin production, we preclude that a receptor–ligand mediated desensitization in WT NK cells could be mechanistically involved. Based on previous studies, we would expect that reduced desensitization results in a more global hyper‐responsiveness such as increased IFN‐γ production [12, 15, 16], which was unaltered in our setting.

The molecular mechanism how NKG2D blocks perforin production remains to be elucidated. Future experiments should focus on the discrepancy between ex vivo sorted and IL‐2 activated NK cells. The kinetics of the observed changes in perforin production should be analyzed in detail and upon different stimuli. Next generation sequencing such as RNA‐Seq or ATAC‐Seq experiments combined with quantitative Mass‐Spec analysis could help to reveal mechanistic insights.

While we observed enhanced perforin expression in IL‐2 expanded NKG2D^ΔNK^ NK cells, killing of TAP‐1 deficient RMA‐S cells was comparable between NKG2D^ΔNK^ and NKG2D^fl/fl^ NK cells both in vitro and in vivo. This might be explained by unchanged expression of granzyme B. Perforin and granzyme B act together; perforin is the pore forming protein while ganzyme B initiates the apoptotic cascade [32, 33]. In vitro cytotoxicity assays proved that NKG2D is involved in the lysis of RMA‐Rae1 cells, YAC‐1 and *v‐abl* ^+^ leukemic cells. The NK cell and NKG2D‐dependent tumor growth control of RMA‐Rae1 cells served as control for our experimental setting in vivo. As YAC‐1 and *v‐abl^+^* cells express additional ligands for the activating receptors DNAM1, our data highlight that engagement of DNAM1 is not sufficient to compensate for the loss of NKG2D in vitro. Despite ligand expression, which results in differences in in vitro cytotoxicity assays, the subcutaneous tumor growth of *v‐abl^+^* cells and the progression of Abelson‐induced leukemia was unaltered irrespective of whether NK cells express NKG2D. This further highlights that in vitro data do not always translate into an in vivo setting. We speculate that NKG2DLs might be shed in the *v‐abl* model, a NKG2D‐mediated desensitization could be taken into account and/or the increased perforin levels could partially compensate for the loss of NKG2D. Further experiments are necessary to study this complex situation.

We observed a slightly increased rejection of B2m^−/−^ cells in vivo despite unaltered expression of Ly49C/I. Although B2m^−/−^ splenocytes express low levels of NKG2DLs, the outcome of these experiments was unanticipated. While NKG2D‐deficient NK cells show an enhanced clearance of B2m^−/−^ cells, the in vitro killing and in vivo surveillance of RMA‐S cells was unaltered. Both B2m^−/−^ cells and RMA‐S cells are recognized and killed via missing self but only B2m^−/−^ cells are differently rejected in the absence of NKG2D. While the cause for this discrepancy remains unclarified, the differences in the route of injection and the time frame between the in vivo tumor models, and the complete absence of NKG2DLs on RMA‐S cell need to be taken into account.

In summary, this study sheds light on a novel transcriptional regulation of perforin downstream of NKG2D but supports the theory that NKG2D plays a dual role in the cytotoxic potential of NK cells. Many open questions remain till we ultimately understand the molecular mechanisms how NKG2D shapes tumor surveillance and to predict the disease progression in the presence or absence of this activating receptor. This complexity has to be considered when NK cells are exploited as an immunotherapeutic tool. Our mouse model will serve as a useful tool to further study the effects of intrinsic loss of NKG2D in mature NK cells.

## Materials and methods

### Mice and cell lines

NKG2D^fl/fl^ mice were kindly provided by Bojan Polić [29] and crossed to *Ncr1‐iCreTg* mice [19]. NKG2D^Δ/Δ^ *Ncr1‐iCre^Tg^* (NKG2D^ΔNK^) and NKG2D^fl/fl^ littermates were bred on C57BL/6N background. B2m^−/−^ *(B2M^−/−^; B6.129‐B2m^tm1Jae^*) mice [34] were kindly provided by Wilfried Ellmeier (Medical University of Vienna, Austria). All mice were bred and maintained at the University of Veterinary Medicine Vienna under pathogen‐free conditions according to FELASA guidelines. All experiments were performed with gender‐ and age‐matched 8–12 wk old animals. Animal experiments were approved by the Ethics and Animal Welfare Committee of the University of Veterinary Medicine Vienna and granted by the national authority (Austrian Federal Ministry of Science and Research) according to §§ 26ff. of Animal Experiment Act, Tierversuchsgesetz 2012 – TVG 2012, under licenses BMBWF‐68.205/0174‐V/3b/2018, BMBWF‐68.205/0178‐V/3b/2018, and BMWFW‐68.205/0093‐WF/V/3b/2015 and were conducted according to the guidelines of FELASA and ARRIVE.

RMA [35], RMA‐S [36], RMA‐RAE1 [37], RMA‐MULT1, and YAC‐1 [38] cells were cultured in RPMI‐1640 (Sigma) complete medium containing 10% FCS (Bio & Sell), 50 µM β‐mercaptoethanol (Sigma), 100 U/mL penicillin, and 100 µg/mL streptomycin (Sigma). *V‐abl* ^+^ transformed cell lines used in this study were derived from C57BL/6N mice and cultured in RPMI‐1640 complete medium [39].

### NK cell isolation, expansion, and stimulation

NK cells were enriched from spleen single cell suspensions using DX5‐labeled MACS beads according to manufacturer's instructions (Miltenyi Biotec) and expanded in RPMI‐1640 complete medium supplemented with 5000 U/mL rhIL‐2 (Proleukin, Novartis) for 7 days. For experiments using ex vivo sorted NK cells, splenocytes were enriched using DX5‐labeled MACS beads followed by sorting for CD3^−^ NK1.1^+^ NKp46^+^ cells on a BD FACS Aria III. Purity of the cells post sorting was ≥99%. Cell sorting experiments were performed according to the guidelines in immunological studies [40]. NK cell stimulations were performed with 5000 U/mL rhIL‐2 ± 5 ng/mL rmIL‐12 (R&D Systems), ±50 ng/mL rmIL‐15 (PreproTech), ±5 ng/mL rmIL‐10 (R&D), or 100 U/mL rmIFN‐ß (Sigma). For receptor‐crosslinking, test tubes were coated with 100 µL of 1–10 µg/mL anti‐NK1.1 antibody (PK136), 1–10 µg/mL anti‐NKp46 (29A1.4), or 10 µg/mL aDNAM1 (10E5) one day before the experiment. Splenocytes were isolated and 10% of the single cell suspension obtained was seeded in the previously coated tubes in RPMI‐1640 complete medium. Brefeldin A solution was added upon 1 h of stimulation/incubation at 37°C. After additional 3 h of incubation at 37°C, the cells were stained with CD3, NK1.1, NKp46, CD49b followed by IFN‐γ and GZMB for flow cytometry. For pSTAT stainings, splenocytes were incubated with 5000 U/mL IL‐2, 5 ng/mL rmIL‐10, or 100 U/mL rmIFN‐β for 20 min. pSTAT stainings in IL‐2 expanded NK cells were performed after 2 h IL‐2 starvation and 20 min re‐stimulation with the respective cytokines.

### In vitro cytotoxicity assays

For in vitro cytotoxicity assays NK cells were DX5‐MACS enriched and expanded for 7 days in IL‐2 as described above. NK cells were mixed at the indicated effector:target ratios with CSFE (Molecular Probes, CellTrace^TM^ CSFE Cell Proliferation Kit) labeled target cells. After 4 h incubation at 37°C the specific lysis was assessed by flow cytometry using SYTOX^TM^ Blue Dead Cell Stain (Invitrogen^TM^) to quantify lysed target cells. Percentage of specific lysis was calculated as follows: [% Sytox^+^ CFSE^+^ cells after co‐incubation with NK cells] – [% Sytox^+^ CFSE^+^ cells without addition of NK cells (spontaneous lysis control)].

### Western blot analysis

Cell lysis, SDS‐PAGE, and Western Blots were performed as described previously [23]. Detection of chemiluminescence was performed using Clarity^TM^ Western ECL substrate (BioRad) by ChemiDocT XRS+ Molecular Imager (BioRad) and analyzed by using the Image Lab software (BioRad). Antibodies against perforin (#3693), pSTAT1^Y701^ (#9167S), pSTAT1^S727^ (#9177), STAT1 (#9172), pSTAT3^Y705^ (#9131), STAT3 (#12640), GrzmB (#4275S), rabbit IgG (HRP linked, #7074S), and mouse IgG (HRP linked, #7076S) were purchased from Cell Signaling Technology. Antibodies against β‐actin (#47778), HSC‐70 (#7298), and STAT5 (#836) were purchased from Santa Cruz Biotechnology. Antibodies against pSTAT4^Y693^ (38/p‐Stat4), STAT4 (8/Stat4), and pSTAT5^Y694^ (47/Stat5(pY694)) were purchased from BD Biosciences.

### Quantitative Real‐time PCR

For RNA preparations of IL‐2 expanded NK cells, cell pellets were resuspended in 1 mL Trizol (PeqGOLD TriFast^TM^, Peqlab) and RNA was isolated according to the manufacturer's protocol. RNA from ex vivo sorted NK cells was isolated using the RNeasy MiniKit (Quiagen) according to the manufacturer's protocol. RNA concentrations were measured using Nanodrop One^C^ (Thermo Scientific). One microgram RNA was reverse transcribed into cDNA using the iScript^TM^ cDNA Synthesis Kit (BioRad). For qRT‐PCR, 10 ng cDNA were used for IL‐2 expanded NK cells and 20 ng for ex vivo derived NK cells. The PCR reaction was performed on a C1000 Touch Thermal Cycler CFX96 Real Time System (BioRad) using SsoFast^TM^ EvaGreen^®^ Supermix (BioRad). The following PCR protocol was used: 3 min 95°C – 40× [10 s 95°C – 30 s 57°C] – 1 min 95°C – 1 min 57°C. The following primers were used: *GrzmB* fwd: CCAATCAGATATGTGCGGG, rev: GGAAACTATGCCTGCAGCC; *Prf1* fwd: GATGTGAACCCTAGGCCAGA, rev: GGTTTTTGTACCAGGCGAAA; *Ifng* fwd: AAGTGGCATAGATGTGGAAG, rev: GAATGCATCCTTTTTCGCCT; *Ube2d2* fwd: AGGTCCTGTTGGAGATGATATGTT, rev: TTGGGAAATGAATTGTCAAGAAA.

### In vivo models

In the A‐MuLV model newborn mice were injected with 100 µL of replication‐incompetent ecotropic retrovirus encoding for *v‐abl* by intraperitoneal injection as described previously [27]. Mice were checked daily for disease onset. At signs of disease manifestation mice were sacrificed, body and spleen weight was determined, and spleen, BM, and blood were analyzed for the infiltration of B cells (CD3^−^CD19^+^).

For *v‐abl^+^#2*, RMA‐S and RMA‐Rae1 tumor models, 10^6^ cells were injected subcutaneously into both flanks of mice and tumor growth was controlled daily. Mice were sacrificed after 10–14 days and tumor weight was determined. For flow cytometry subcutaneous tumors were cut in 2–5 mm^2^ pieces and digested using the gentleMACS^TM^ *Octo Dissociator* (Miltenyi Biotec) with digestion buffer containing 1mg/ml Collagenase D (Roche) and 20 µg/mL DNAse I (Roche).

For the B2m^−/−^ rejection assay NKG2D^ΔNK^ and NKG2D^fl/fl^ littermates were injected intravenously with a 1:1 mixture of 5 × 10^6^ 5 µM CSFE (CSFE^high^) labeled C57BL6/N splenocytes and 5 × 10^6^ 0.5 µM CSFE (CSFE^low^) labeled B2m^−/−^ splenocytes. After 16 h, spleens of injected mice were taken and analyzed for CSFE^high^:CSFE^low^ ratio and rejection capacity was calculated.

### Flow cytometry

Flow cytometry experiments were performed according to the guidelines in immunological studies [40]. Single cell suspensions of BM or spleen were prepared. For detection of IFN‐γ and granzyme B, the BD Cytofix/Cytoperm^TM^ Fixation/Permeabilization Solution Kit (BD Bioscience) was used according to manufacturer's instructions.

For flow cytometric intracellular perforin and pSTAT stainings, splenocytes were stimulated, stained for CD3, NK1.1, and NKp46, fixed with 2% paraformaldehyde (Sigma), and permeabilized with ice‐cold 90% methanol before intracellular stainings.

The following antibodies (clones) were purchased from eBioscience/Invitrogen^TM^: CD3 (17A2), CD11b (M1/70), CD27 (LG.7F9), CD107a (1D4B), CD122 (5H4), DNAM1 (10E5), CD49b (DX5), Gr‐1 (RB6‐8C5), granzyme B (NGZB), IFN‐γ (XMG1.2), Ly49A (A1(Ly49A)), Ly49G2 (4D11), Ly49D (4E5), NKG2A (16a11), NKG2A/C/E (20d5), NKG2D (CX5), NKp46 (29A1.4), NK1.1 (PK136), and Ter119 (TER‐119). The following antibodies were purchased from BD Pharmigen^TM^: CD19 (1D3), B220 (RA3‐6B2), pSTAT1 (4a), pSTAT3 (4/P‐STAT3), and pSTAT5 (47/STAT5(pY694)). The following antibodies were purchased from Biolegend: perforin (S16009A), isotype IgG2aκ (RTK2758). The following antibodies were purchased from R&D Systems: RAE‐1 (186107), MULT1 (237104), and isotype IgG2A (20102). Ly49C/I (REA253) was purchased from Miltenyi Biotec. SYTOX^TM^ Blue Dead Cell Stain (Invitrogen^TM^) was used to detect apoptotic cells. Flow cytometry experiments were performed on a BD FACSCanto II (BD Bioscience) and analyzed using FlowJo V10 software.

### Microarray analysis

Raw Affymetrix HG‐U133_plus_2.0 microarray data from dataset GSE12198 were obtained from ArrayExpress database. Data normalization, background correction, and log_2_ transformation were performed using the frozen robust multiarray (fRMA) algorithm and R 3.4.2 software [41]. Visualization of log_2_ transformed gene expression data was done using ClistVis tool [42].

### Statistical analysis

Unpaired one‐ and two‐sided *t*‐test and log‐rank test were performed using GraphPad Prism^®^ (GraphPad Software). The significance is indicated for each experiment.

## Author's contribution

D.P., K.K., J.L., V.K., I.M., A.W.S., N.L, and D.G. performed the research; D.P, A.W.S., E.M.P., and D.G. established methods; D.P., A.W.S, E.M.P., D.G., and V.S. designed the research and analyzed data; G.H. analyzed the microarray data and provided the figure and helped writing the manuscript. B.P. and V.S provided material. D.P., D.G., and V.S. wrote the manuscript.

## Conflict of interest

The authors declare no commercial or financial conflict of interest.

**Abbreviations**
NKG2D: NK group 2D
NKG2DL: NKG2D ligand
MULT‐1: mouse UL16‐binding protein‐like transcript 1
RAE‐1: retinoic acid early inducible 1

## Supporting information

Supporting Information
