# Supplementary material for: Loss of NKG2D in murine NK cells leads to increased perforin production upon long‐term stimulation with IL‐2
Source: Eur J Immunol. 2020 Feb 20;50(6):880–90. doi: 10.1002/eji.201948222 (PMC7318224; doi:10.1002/eji.201948222)
Supplement: Supplementary file 1 — Supporting Information [file EJI-50-880-s001.pdf]

Supplementary Figure 1

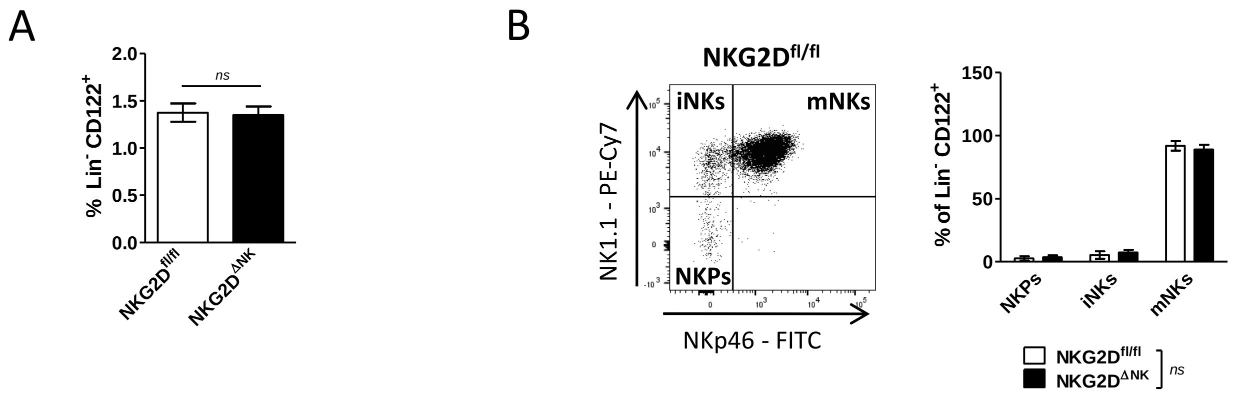

Supplementary Figure 1. NK cells in the bone marrow of NKG2D$^{\Delta NK}$ mice are comparable to controls. (A) Percentage of NK cells in the bone marrow defined as CD3$^-$CD19$^-$Gr1$^-$Ter119$^-$(Lin$^-$)CD122$^+$ cells is shown. (B) Dot plot showing the gating strategy to analyze NK cell development in the bone marrow. First, NK cells were gated on Lin$^-$CD122$^+$ and consecutively divided into NK precursors (NKPs: NK1.1$^-$NKp46$^-$), immature NK cells (iNKs: NK1.1$^+$NKp46$^-$) and mature NK cells (mNKs: NK1.1$^+$NKp46$^+$). Graph shows mean ± SD (n=10 per genotype pooled from 3 independent experiments). Gating of strategy of Lin$^-$CD122$^+$ is shown in Figure S6C.

Supplementary Figure 2

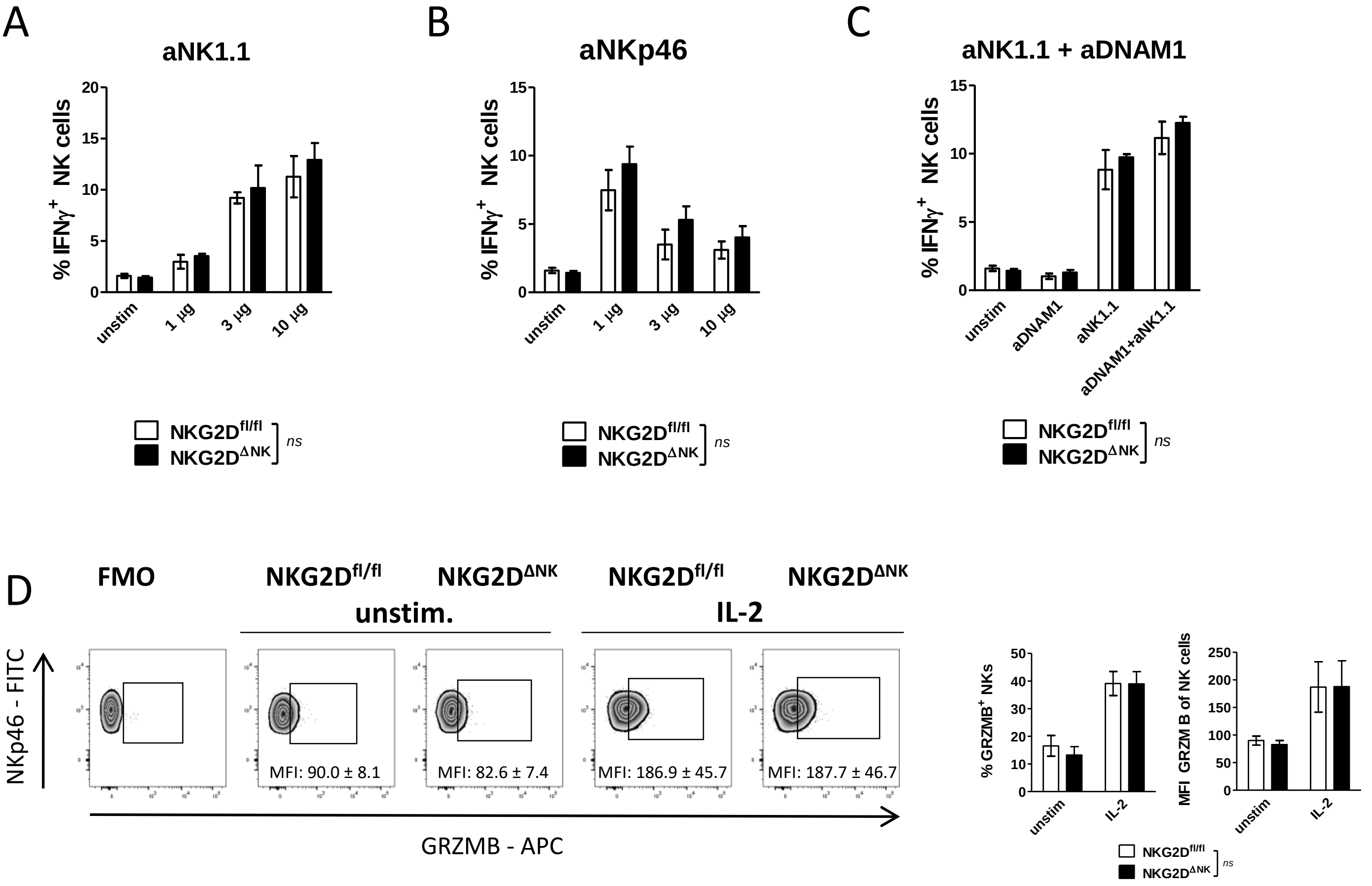

Supplementary Figure 2. NKG2D deficient NK cells show unaltered IFNγ and granzyme B production. Freshly isolated splenocytes of NKG2D$^{\Delta NK}$ or NKG2D$^{\Delta fl/fl}$ mice were incubated with increasing concentrations of either (A) immobilized aNK1.1, (B) aNKp46 or (C) aDNAM1 and aDNAM1 + aNK1.1 or media alone (unstim.) for 4 hours as indicated. Proportions of IFNγ+ cells gated on CD3-NKp46+ for aNK1.1 and aDNAM1 stimulation and CD3-NK1.1+ for aNKp46 stimulation were analyzed via flow cytometry. Results indicate mean ± SD (n ≥ 3 per genotype, unpaired t-test). (D) Freshly isolated splenocytes were stimulated with IL-2 or media alone for 4 hours to assess granzyme B levels via intracellular flow cytometry. Plots show representative results. Bar graphs indicate mean ± SD (n=6 per genotype, pooled from two independent experiments; two-sided unpaired t-test). Gating strategy is shown in Figure S6A.

Supplementary Figure 3

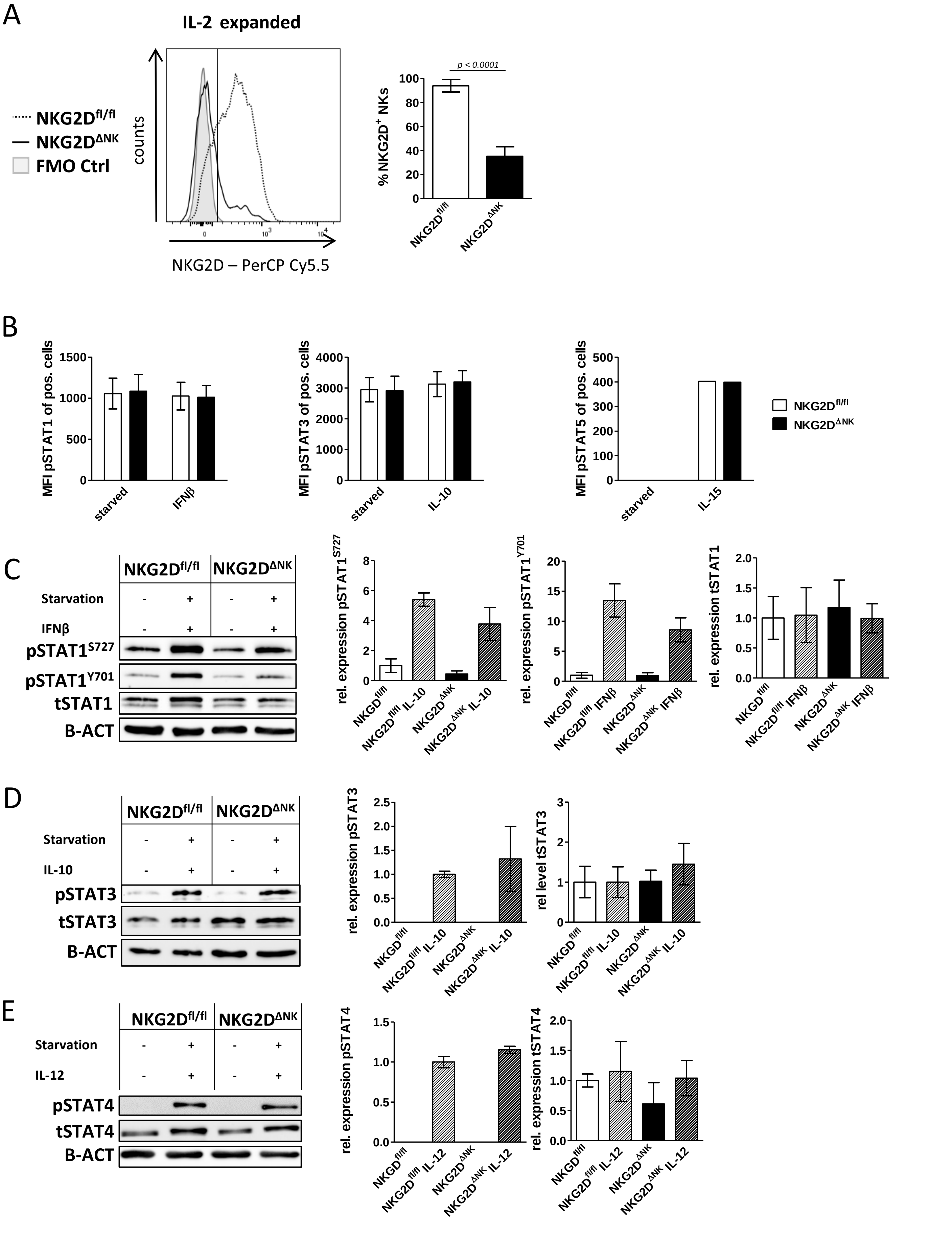

Supplementary Figure 3. IL-2 expanded NKG2D-deficient NK cells have unaltered STAT activation. (A) NKG2D expression on IL-2 expanded NK cells upon 7 days of culture. Representative histogram is shown. Bar graph shows mean ± SD (n = 6 per genotype, pooled from 3 independent experiments; tow-sided unpaired t-test). (B) Activation of STAT1, STAT3 and STAT5 was assessed in IL-2 expanded NK cells. NK cells were starved from IL-2 for 2 hours before re-stimulation for 20 minutes with indicated cytokines. Mean fluorescence intensity (MFI) of the positively stained population is represented in bar graphs. Graphs represent mean ± SD of one representative experiment (n=2 experiment and 3 technical replicates for pSTAT1 and pSTAT3; two-sided unpaired t-test was used for statistical analysis; n=1 for pSTAT5). (C-E) pSTAT and total STAT protein levels were determined from expanded NK cells or from 2 hours IL-2 starved NK cells upon 15 minutes re-stimulation with IFNβ (pSTAT1), IL-10 (pSTAT3) or IL-12 (p-STAT4). Bar graphs show quantification of protein expression normalized to the loading control (β-actin) and relative to total STAT or pSTAT expression of NKG2Dfl/fl NK cells. Western blot (C) was performed once; (D and Eshows representative blots of two independent experiments. Bar graphs represent mean ± SD of one representative experiment (technical triplicates, two-sided unpaired t-test was used for statistical analysis).

Supplementary Figure 4

A

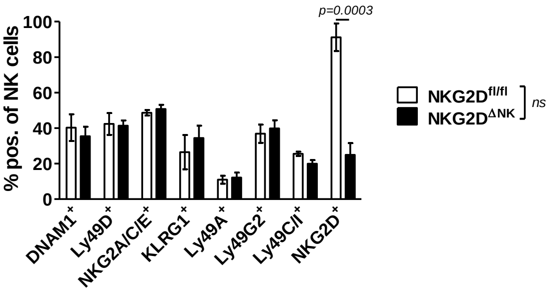

B

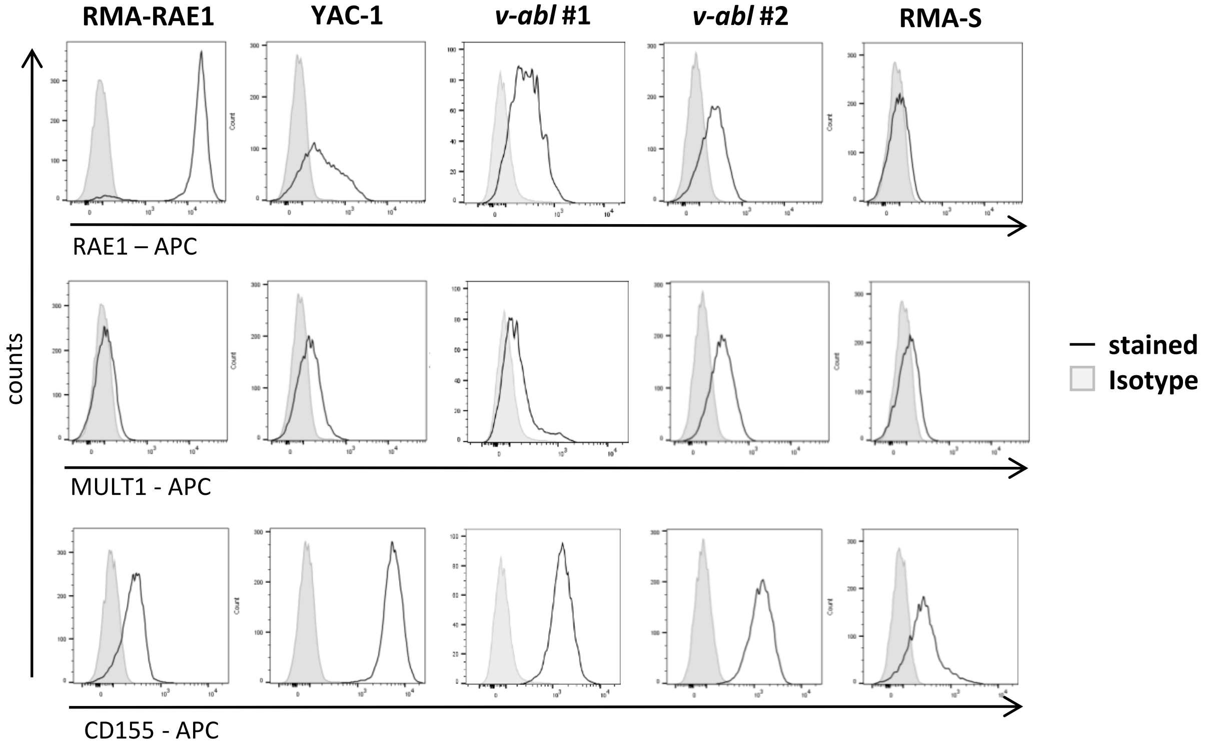

Supplementary Figure 4. Surface receptor expression in IL-2 expanded NKG2D-deficient NK cells is unaltered.

(A) IL-2 expanded NK cells were examined for surface receptor expression via flow cytometry. Bar graphs show mean ± SD of percentage of positively stained NK cells (n=4 per genotype; two-sided *t*-test). (B) Diverse NK target cell lines were screened for expression of NK cell-activating ligands RAE1, MULT-1 and CD155. Representative histograms from 3 independent experiments are shown. Gating strategy is shown in Figure S6B.

Supplementary Figure 5

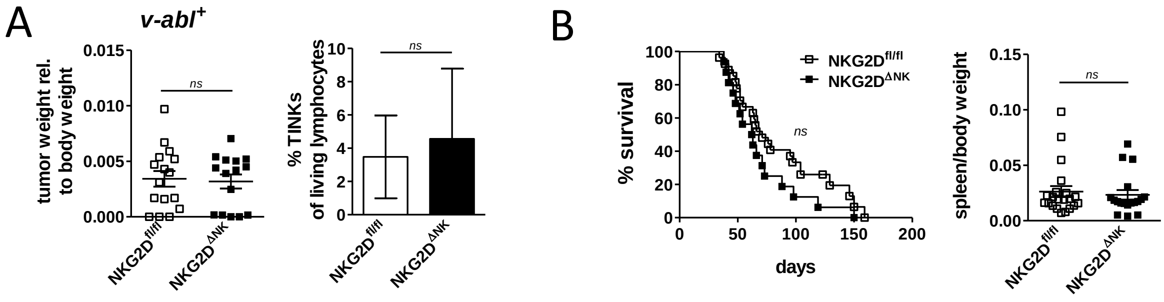

Supplementary Figure 5. Tumor immunosurveillance of *v-abl*$^{+}$ leukemic cells is unaltered in NKG2D$^{\Delta NK}$ mice.

(A) Mice were injected subcutaneously with *v-abl*$^{+}$ cells. Tumor to body weight ratio was determined at endpoint and infiltrating NK (CD3^{-} NK1.1^{+} NKp46^{+}) cells were analyzed via flow cytometry. Bar graphs represent mean ± SD (n = 16 per genotype pooled from two independent experiments; two-sided unpaired *t*-test). (B) Kaplan-Meier plot of NKG2D$^{\Delta NK}$ (n = 16) and NKG2D$^{fl/fl}$ (n = 27) mice injected subcutaneously with a *v-abl* encoding replication-incompetent ecotropic retrovirus 24-48 h after birth inducing a slowly progressing pro-B cell leukemia (data pooled from two independent experiments). Animals were euthanized at the first sign of health detraction. Spleen to body weight ratio of diseased NKG2D$^{\Delta NK}$ or NKG2D$^{fl/fl}$ mice. All graphs show mean ± SD.

Supplementary Figure 6

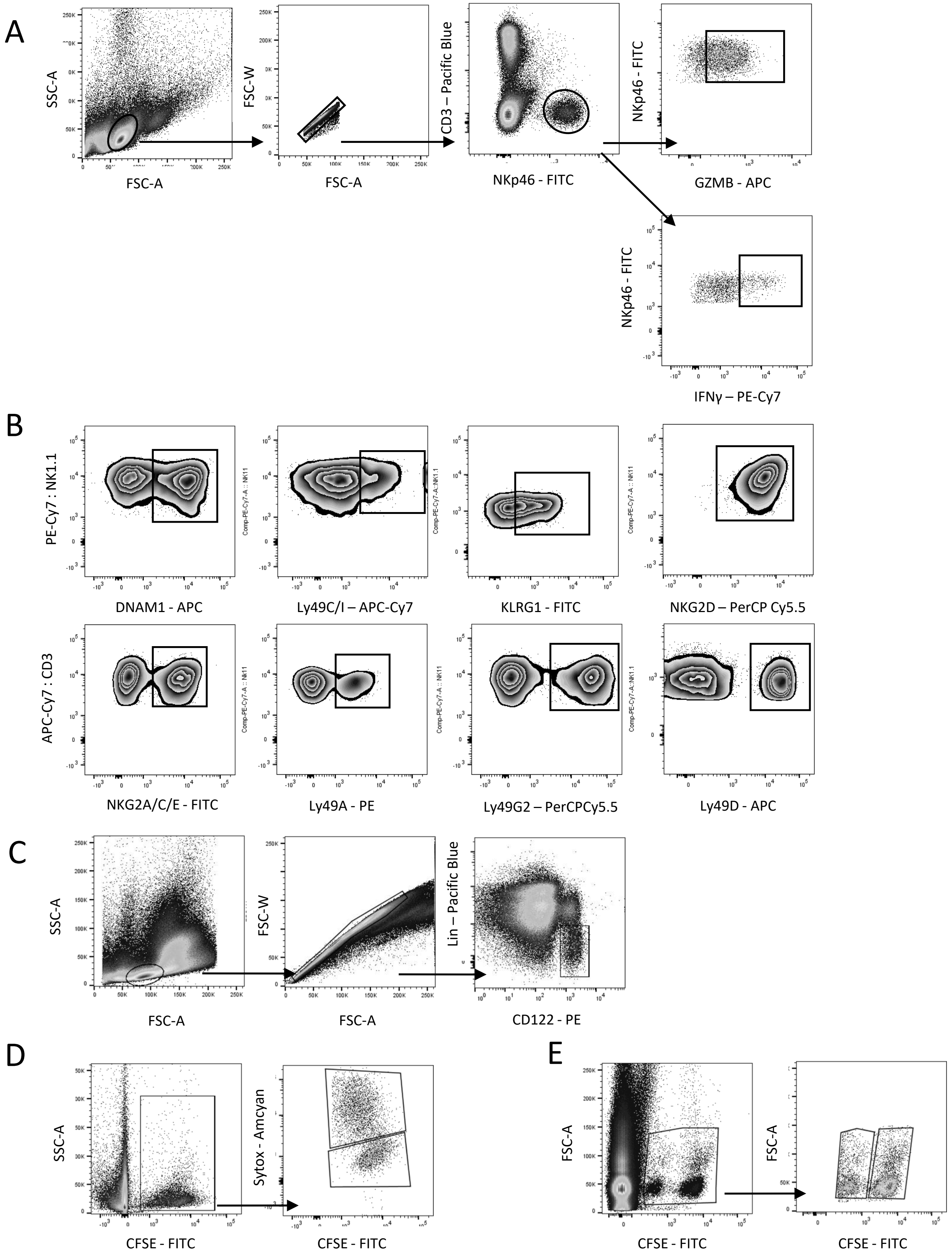

Supplementary Figure 6: Gating Strategies

Gating strategy for NK cells in spleen and tumor are shown. Starting with a lymphocyte gate, we continue to gate on single cells. we define NK cells as $CD3^{-}NKp46^{+}$. From this gate we can either look at (A) intracellular expression of IFNγ and granzyme B or (B) activating and inhibitory receptors on the NK cell surface. (C) Gating strategy for NK cells in the bone marrow is shown. Starting with a lymphocyte gate, we continue to gate on single cells, from there we gate on $Lin^{-}CD122^{+}$ as starting gate to differentiate the developmental stages of NK cells (see Figure S1). Examples of the gating strategy for (D) *in vitro* cytotoxicity assays and (E) *in vivo* $B2m^{-/-}$ rejection assay.
